# A Disability Act? The Vaccine Damage Payments Act 1979 and the British Government’s Response to the Pertussis Vaccine Scare

**DOI:** 10.1093/shm/hkv140

**Published:** 2016-08-04

**Authors:** Gareth Millward

**Keywords:** vaccination, disability, policy, social security, public health

## Abstract

The Vaccine Damage Payments Act 1979 provided a lump-sum social security benefit to children who had become severely disabled as a result of vaccination. It came in the wake of a scare over the safety of the whooping cough (pertussis) vaccine. Yet very little has been written about it. Existing literature focuses more on the public health and medical aspects of both the Act and the scare. This article uses material from the archives of disability organisations and official documents to show that this Act should be seen as part of the history of post-war British disability policy. By framing it thus, we can learn more about why the government responded in the specific way that it did, as well as shed new light on public attitudes towards vaccination and disability.

In the mid-1970s, a group of British parents claimed that their children had become disabled as a result of government-recommended vaccinations. Although their complaints covered a range of diseases, it was the whooping cough—pertussis—vaccine that captured the public imagination. Sections of the medical community backed the parents’ position, and the vaccination rate for pertussis plummeted. The confusion was such that when the government was advised by its own expert bodies that a major publicity campaign was necessary to avoid a whooping cough epidemic, it declined to do so until it had received the results of epidemiological studies into the safety of the vaccination programme. In an attempt to restore confidence, the Labour government forced through legislation that would provide payments of £10,000 to those who could show that their children had been damaged. But this was too late to avoid a pertussis epidemic in the winter of 1978/79.

The Department of Health and Social Security (DHSS) engaged in a two-pronged defence of the vaccination programme. First, its advisory bodies the Committee on the Safety of Medicines and the Joint Committee on Vaccination and Immunisation (JCVI) reviewed the evidence on the safety and efficacy of the pertussis vaccine. Second, to restore public trust it passed the Vaccine Damage Payments Act 1979 to provide social security payments to families of damaged children. The former has received attention from historians and researchers of public health. The latter, however, has been largely ignored, or presented as part of the medical establishment’s response to the ‘pertussis vaccine scare’. As this article demonstrates, such analyses overlook the crucial influence of contemporary political factors. In particular, developments in disability policy and the position of disabled people fuelled and, in turn, provided some of the tools for responding to the crisis.

## Disability, Vaccination and Social History

The current historiography on the whooping cough crisis of the 1970s and early 1980s has focused on the public health ramifications. This has meant that the primary source materials and analytical focus have been predominantly medical or concerned with the minutiae of public health policy. Yet the events are better explained through the prevailing political and social context. This is by no means a new approach to the history of medicine. Porter and Porter, for example, have shown that opposition to compulsory smallpox vaccination in the nineteenth century was tied into a number of cultural attitudes towards poverty and pauperism, as well as scepticism over the truth claims of the emerging fields of epidemiology and public health.^1^ On the disability side, Borsay has shown how disabled people were institutionalised during the modern period; but at the same time, many participated in public and private pursuits. This has provided a richer view of how health status and concepts such as disability and capacity were understood in modern British society.^2^ By highlighting the disability aspects of the Vaccine Damage Payments Act 1979, we can better understand reactions to both public health and disability issues in the post-war era. The Act was explicitly framed using disability legislation, telling us much about the legal framework derived from a definition of disability that had evolved from earlier decades. The catalyst was a medical scandal, born in the wake of the thalidomide crisis and played out in the political discourse of the period. This is, therefore, an opportunity to see public anxieties over acute and chronic health concerns in action.

Work has been done on public and organised opposition to smallpox vaccination in the nineteenth century, and has included debates over class, religion, scientific consensus and the rights of individuals versus the collective.^3^ Many of these themes continued into the twentieth century, as has been shown with other emerging immunisation techniques and how they were accepted by their target population.^4^ However, immunisation in post-war Britain is much less studied from a historical perspective.^5^ For whooping cough in particular, we are left with mainly medical and epidemiological analyses rather than political or social history. These locate the controversy almost exclusively within public health practice and policy. This ignores much of the contemporary political climate, including the growing disability movement, sweeping reforms to social security benefits for disabled people, the legacy of the recent thalidomide scandal and a deepening financial crisis. As a result, the scare has been somewhat dehistoricised and placed in the context of proceeding. Broadly, this has created two types of analysis. The first uses the pertussis vaccination and incidence data from the 1970s and 1980s to show that the epidemics of 1979 and 1982 were much larger than at any point before or since the introduction of a routine diphtheria-tetanus-pertussis (DTP) vaccine in 1957.^6^ The scare is therefore presented as a cautionary tale of the risks of allowing fear of vaccine safety to grow amongst the public, as well as evidence for the efficacy of mass vaccination programmes. The second type of analysis draws parallels with the later MMR controversy of the late 1990s and early 2000s.^7^ This is problematic, because both scares were produced in very specific historical conditions. Instead, they are thought equivalent because the central concern of these studies is how to measure the effects of declining vaccination rates. Both these forms of analysis gloss over the political and social context of the period, and take as granted the hindsight that the pertussis vaccination was declared safe in the early 1980s.^8^ Further, they tend to disregard the historical importance of the scare in its own right in favour of wider practical questions about public health and vaccination. For public health practitioners, the scandal represents ‘bad science’, to borrow a term, in a world that is more prone to focus on the ‘lesson of history’ for concrete action rather than to understand the motives of policy actors within their own specific historical context.^9^

For these reasons, it is important for historians to look beyond the medical sphere. As Drakeford and Butler have shown, ‘scandals’ such as this are manufactured to a certain extent.^10^ The mere existence of morally offensive action is not enough; it needs to be articulated through public discourse.^11^ This is true not just of a scandal, but also of the specific responses that are chosen by policy makers. Kingdon has noted that policy action requires the confluence of a perceived problem, the political will to act, and the technical capacity to respond.^12^ That is to say, it was neither inevitable that the knowledge of vaccine damage would turn into the scandal that it did; nor that the Vaccine Damage Payments Act would be one of the policy results. The pertussis scare occurred at a crucial time in disability politics in which both Labour and Conservative governments had enacted a range of policies aimed at improving the lives of disabled people. Disability was being seen as a social issue as well as (if not instead of) a medical one; and it had become a branch of policy with its own machinery for creating solutions to policy problems.^13^ Further, a voluntary organization in the form of the Association of Parents of Vaccine Damaged Children was able to convince medical, state and private institutions that their favoured solution to the problem—statutory compensation—was the morally correct course of action.

The Vaccine Damage Payments Act has received relatively little scrutiny of this type partly because it is, historically speaking, a recent event. The traditional ‘thirty-year rule’ at The National Archives means that much of the material upon which this article is based has only been publicly available for a few years. Moreover, disability histories are themselves relatively new.^14^ Histories of welfare provision have tended to focus on the wider ‘rediscovery of poverty’ and the politics of ‘consensus’ in the 1960s and 1970s which provided a fertile environment for extending the welfare state to groups excluded from the post-war settlement.^15^ Recent work has shed light on the lives of disabled people and the various institutions which governed their lives.^16^ This has begun to include discussions of the social construction of impairment and the differing experiences of disabled people.^17^ This journal has also shown a growing interest in the implications for the social history of medicine.^18^ But this relatively new endeavour has only just begun to investigate the period after 1970. As such, there is very little on the two big disability compensation crises of the decade—thalidomide and vaccine damage. Memoirs and biographical material of the major players have been produced in which the campaigners narrate their side of the story, yet there is almost nothing on how these affected the position of ‘disability’ as a social and legal concept.^19^ Jameel Hampton has discussed the quandary confronted by the Disablement Income Group (DIG) when faced with the claims by the thalidomide parents, but by ending his study of British disability policy in 1975 he does not tackle vaccine damage.^20^ Similarly, Claire Sewell’s work on the parents of disabled children is analysed in the wake of the thalidomide crisis, but does this to draw wider conclusions about parenting, childhood and the concept of ‘the carer’.^21^ Other than Jeffrey Baker’s overview of the pertussis vaccine controversy and its effects on American anti-vaccination campaigns, there is no investigation into the primary material surrounding the Vaccine Damage Payments Act and its significance in British disability policy.^22^

This article argues that by placing the Act in this wider political and social discussion, historians can learn more about why the government responded as it did to the whooping cough scare. Campaigners successfully used the tactics of other voluntary organizations to press their case to the public. They were able to draw on both the successes of the poverty lobby over the 1960s and recent health scandals which remained fresh in the memory. Importantly, the specific framework of the Act drew heavily on existing disability policies and definitions. It is only by moving beyond the existing medical narratives that we can access this history. To go further, even though the Act has not been part of the traditional narrative of disability policy, we can understand much about government attitudes towards disability through the provisions contained within the Act.

## The Association of Parents of Vaccine Damaged Children

The Association of Parents of Vaccine Damaged Children (hereafter the Association) was formed in 1973 by two mothers who blamed their children’s brain damage on the poliomyelitis vaccine. A piece in the *Birmingham Post* in June 1973 on the subject of vaccine damage included a call from Rosemary Fox and Renee Lennon to establish a new society.^23^ By the time their story was published in a *Guardian* article in August, this society was calling itself the Association.^24^ Fox and the Association became the public face of the campaign to provide compensation for victims of vaccine damage.

In many ways, the Association drew on the tactics and successes of earlier disability organisations. Unlike others in the ‘poverty lobby’ or ‘welfare rights’ sphere, it was largely a single-issue group. DIG became the first pan-impairment disability organisation to lobby central government in 1965.^25^ Other voluntary organisations at this time concerned themselves with specific impairments or groups of conditions—notable examples being the Spastics Society and MENCAP—or were charities providing care for disabled people—such as Leonard Cheshire.^26^ DIG’s concern was wide-ranging, and included a complete reformulation of the social security system with regard to disabled people. It established a campaign for a National Disability Income, an ideal social security benefit that would compensate disabled people for the lost earnings and additional costs associated with living with single or multiple impairments.^27^ In 1974, the Disability Alliance would promote a similar campaign led by prominent sociologist and poverty campaigner Professor Peter Townsend.^28^

The Association, however, focused solely on the issue of compensation for vaccine damaged children. This demand for special treatment was politically problematic. Both DIG and the Disability Alliance argued against the system, which had developed after the Second World War and gave preferential treatment to certain categories of disabled people. Claimants with National Insurance records or those injured in industrial accidents or the armed forces were entitled to higher levels of benefit; while married women qualified for no benefits at all. Moreover, the system was designed to provide temporary cover for sickness and unemployment rather than the specific effects of chronic illness. This approach remained most prominent throughout the 1970s,^29^ but it was already coming under scrutiny from more radical opponents. Social security was seen by the Union of the Physically Impaired Against Segregation as a symptom of disability, not the root cause of why disabled people were discriminated against. Drawing on feminist and black critiques of sexism and racism, it directly challenged the dominant ‘medical model’—whereby most political and cultural institutions defined disabled people by medical diagnoses, or what was ‘wrong’ with their bodies and minds. A new social model was proposed in which people were said to be disabled by society.^30^ For example—a person is not disabled because they cannot climb stairs; they are disabled because buildings are designed for an assumed level of capacity in which everyone can climb stairs. Thus, the focus of disability policy should not be on manipulating the individual to walk (necessarily), but should instead look to install escalators and lifts in public buildings so that everyone has access to core services.^31^ While these groups did not gain significant public attention until the 1980s, it must be noted that the Association’s focus on the specific medical problems of their members’ children went against many of the political developments of the decade.

Until the financial crises of the Callaghan years restricted government expenditure, the campaigns for disability benefits were largely successful. DIG and the Disability Alliance became frustrated at the slow rate of progress, but after the passing of the Social Security Benefits Act 1975, most of the groups that DIG had campaigned for were now covered by at least some sort of benefit.^32^ The outgoing Wilson government in 1970 attempted to create a limited invalidity pension, but the National Superannuation and National Insurance Bill was lost to the general election. Such was the political consensus on the matter, however, that the new Heath administration quickly established Invalidity Benefit and Attendance Allowance to help unemployed disabled people and the cost of caring for a disabled relative respectively. When it returned to power, Labour created benefits for housewives, the costs of transport and a non-contributory version of Invalidity Benefit in 1975. Campaigners had managed to secure statements from successive Secretaries of State for Social Services that, once the economy recovered, the system would be reformed and improved upon.^33^ For the Association, this meant that it was widely accepted that monetary payments were an important facet of social policy for disabled people; and that there was a growing set of precedents upon which a compensation scheme could be built.

The result of this growing movement was a wider consideration of the needs of disabled people, and parliamentarians were beginning to specialise in this area of policy. This gave the Association the opportunity to build alliances with sympathetic figures in Westminster. In 1969, Alfred Morris introduced the Chronically Sick and Disabled Persons Bill. When it received Royal Assent the following year, it was the first such Act of its kind in the world, giving powers to local authorities to provide services for disabled people.^34^ While it never had the powers of compulsion that Morris and the Bill’s supporters had hoped for, it meant that after the February 1974 General Election Morris became the world’s first Minister for the Disabled.^35^ He had been helped by the creation of the All Party Group on Disablement, founded by Jack Ashley (Labour, Stoke-on-Trent South) and John Astor (Conservative, Newbury).^36^ Ashley was central to the Association’s activities in Westminster. He was also disabled, deaf as a result of an infection he had contracted after surgery on his ears. In 1974 he became Parliamentary Private Secretary to Barbara Castle (Secretary of State for Social Services), which gave the Association an opportunity to speak directly to the head of the DHSS.^37^ Even when Ashley left his post, his relationship with Morris and new Secretary of State David Ennals ensured that the issue remained on the agenda. This is evidenced not only by the correspondence between Ashley and the DHSS during the 1970s but the involvement of the three men in meetings with Conservative ministers over the Vaccine Damage Payments Scheme in the early 1980s.^38^

Most crucially of all, disability was seen as a social category and a matter of policy.^39^ Yet the rhetoric had been based around equal treatment based on need, not on cause of impairment. The Association was trying to argue that it was a ‘special case’.^40^ The recent thalidomide crisis gave campaigners an analogous medical scandal that could be exploited. The *Sunday Times* campaign for full compensation from the drug’s manufacturers had only just concluded, pursued vigorously by Jack Ashley in parliament. In its wake, the Heath government created the Family Fund to provide payments to ‘congenitally disabled children’.^41^ The experience had made the public and medical establishment wary about the dangers that could be posed by drugs and treatments presumed to be safe.^42^ As a result, The Royal Commission on Civil Liability and Compensation for Personal Injury added medical negligence to its remit.^43^ Chaired by Lord Pearson, it was primarily concerned with the current system of accident compensation, including industrial injuries following the earlier Robens Report into Health and Safety.^44^ Thalidomide had also made the parents of disabled children more visible, even if, as Sewell argues, it had not resulted in a fundamental shift in public attitudes or their legal status.^45^ By simultaneously claiming that the Family Fund did not provide adequate coverage, and appealing to the potential for another thalidomide-like scandal, the Association could make its specific claims for compensation.

## The Vaccine Damage Campaign

The Association’s breakthrough in 1973 came as a result of Fox’s campaigning and the recent publication of an article in the *British Medical Journal* (*BMJ*) which argued:

The moral justification for compensation … is based on the social contract. National immunization programmes not only aim to protect the individual but also to protect society. … If individuals are asked to accept a risk (even a very small one) partly for the benefit of society then it seems equitable that society should compensate the victims of occasional unlucky mishaps.^46^

‘Protect[ing] society’ had taken on a dual meaning. In the short-term, vaccination policy had been focused on preventing infectious disease; but it had also come to mean protection from disability. While early public health interventions had focused more on infectious diseases, the relative increase of chronic disease had seen a shift in priorities.^47^ Vaccination was still seen as an important tool against infectious diseases such as diphtheria and tuberculosis, but immunisations against poliomyelitis and rubella were driven by concerns over the public and private costs of disabled children surviving into adulthood. The campaign against rubella, particularly aimed at women of child-bearing age, was regularly cited throughout the 1970s as part of the government’s disability policy.^48^ Similarly, the request for monetary ‘compensation’ was entirely consistent with demands from welfare rights organisations and the recent success of the parents of children affected by thalidomide.

Fox’s daughter, Helen, had received the polio vaccine in the early 1960s and soon afterwards showed signs of brain damage. She began to have fits and, by the time of the campaign, was eleven years old with a diagnosed mental age of three. Believing that the vaccine had caused this behaviour, Fox and the other parents were angered by the attitude of the profession, with many doctors refusing to acknowledge that vaccination could lead to damage at all. They began to collect detailed medical information from Association members to make their case to the medical authorities.^49^ This had been a core tactic among welfare rights organisations. The Child Poverty Action Group and DIG had collected information from members and those it sought to help in order to be able to provide illustrative examples of the difficulties suffered by those who could not access help from the welfare authorities.^50^

The majority view of the medical establishment was that vaccines were safe and effective measures of disease prevention. A large trial of 36,000 subjects in 1957 conducted by the Medical Research Council had shown the pertussis vaccine to be safe and effective, with no cases of brain damage.^51^ But the parents were not ‘fobbed off’ by everyone.^52^ In particular, Professor Gordon Stewart and Dr John Wilson offered their support to the campaign. A letter to *The Guardian* by Drs J. V. T. Gosling and J. H. Moseley alleged that the pertussis vaccine was not effective enough to be worth administering. They also made reference to some cases of brain damage that might be linked to its use.^53^ This was pressed further in 1974 by Wilson and colleagues at Great Ormond Street Hospital, who alleged a link between brain-damaged children and the whooping cough vaccine.^54^ ‘People seem to worry’, Fox told *The Guardian*, ‘doctors in particular, that they may get themselves involved in another highly publicised thalidomide episode’.^55^

It would also not have been the first high-profile instance of damage to children *en masse* from vaccination. The American Cutter Incident, in which thousands of children were injected with live polio virus as the result of a faulty batch of the new Salk vaccine, had occurred less than 20 years previously.^56^ This led the Association to make a tactical decision and focus its efforts on pertussis. Not only did many of its members (around two-thirds) blame the vaccine for their children’s injuries, there was a growing literature that suggested that there was hard evidence for their case.^57^ Professor George Dick, a member of the JCVI, had noted in 1973 that he had evidence for around 80 cases of damage from the vaccine per year.^58^ By 1977, Gordon Stewart’s claims that it was safer to catch pertussis than receive the inoculation reflected how much attention had been brought to the subject in the media and the medical community.^59^ Although there was never consensus that the vaccine was dangerous, enough doubt had been sown for there to be a genuine debate.

Parliamentarians became interested in the crisis, and the Association’s rising profile saw both the Heath and Wilson governments of the mid-1970s forced to refute allegations of medical negligence or a cover up.^60^ A succession of Early Day Motions, signed by dozens of MPs, suggest at least “soft” support for the campaign’s broad goal of providing compensation for accident victims.^61^ Parliamentary questions from many MPs, but particularly Jack Ashley and Robert Adley (Conservative, Bristol North East until February 1974, then Christchurch and Lymington), pressed the government to release more information and to conduct further enquiries and tests into vaccine safety.^62^ In 1977, Ashley referred a complaint to the Parliamentary Commissioner, Sir Idwal Pugh, on behalf of the Association. Fox and Ashley argued that the health services had

failed to make available to parents all the information they should have taken into account before they agreed to have their children vaccinated against whooping cough (pertussis). Mrs Fox added that she felt medical practitioners and health visitors were generally ill-informed about the conditions which made it inadvisable to give pertussis vaccine in the first place (contra-indications). … She considered that the Departments … had a responsibility also to see that everyone involved had adequate information and guidance on the subject.^63^

Pugh’s report was significant in that it brought specific cases to the attention of Parliament and the media. Ashley had chosen the medical stories of four children that, he argued, showed clear signs of contra-indications that were ignored by doctors at the time, leading directly to vaccine damage.^64^ ‘T’ was apparently ‘normal and healthy’ until he received his second dose of DTP and began having fits. The doctor gave the child a third dose regardless of the symptoms, and now he was considered ‘severely brain-damaged and ineducable’. ‘M’ had an epileptic mother, but has ‘begun to deteriorate after immunisation at fourteen months old and by eighteen months had become totally unresponsive’. ‘K’ had been born prematurely, and after receiving her vaccinations had become prone to ‘bouts of screaming’. The final case, ‘R’, had developed a ‘curious jerking of his left arm’ soon after being vaccinated for the first time, but the issue was dismissed as unimportant. After the second dose, the child began to have convulsions. The Commissioner did not agree entirely with the Association’s assertions of cause and effect, and believed that there was enough information about the benefits and risks of vaccination in the broad public health sense. Still, he believed that information on adverse reactions was poor, and argued that parents and doctors should be given better advice on how to spot contra-indications.^65^

## The Government Response

The government resisted early calls for a compensation scheme for the children, despite showing political sympathy with the Association’s cause. Eventually, however, multiple pressure points forced action. Not only had the campaign for compensation gathered pace, it was becoming increasingly clear that the dramatic decline in vaccination rates was likely to lead to an epidemic in 1978 or 1979.

Ennals made an announcement to the House of Commons in February 1977. The JCVI had urged him to begin a publicity campaign, but he opted to wait until he had harder evidence of the vaccine’s safety, on the advice of the Committee for the Safety of Medicines.^66^ In his speech, he expressed sympathy for the parents, but argued that any action on the matter would have to wait for a detailed report from the JCVI on the scientific evidence, and from the Pearson Report on the legal position of any compensatory scheme.^67^ Pearson had been used as a delaying tactic throughout the Association’s campaign. Castle, in her first meeting with Fox in May 1974, had suggested referring vaccine damage to the Royal Commission.^68^ It also allowed the government to stall (and eventually fight off) a case in the European Commission of Human Rights that was being pursued by the Association.^69^

A series of coinciding factors forced the government into action in the summer of 1977. The entire vaccination programme was in crisis. Vaccination rates for whooping cough had declined 59 per cent between 1971 and 1975.^70^ Pearson was due to publish towards the end of the year, and would most likely recommend a compensation scheme. Consumer information group *Which?* had also made its support for compensation public.^71^ Further, Ennals clearly believed that the whole episode was inflicting significant political damage on the Labour Party. Ashley and the Association had successfully argued their case, and the government’s lack of action was ‘undermining our reputation as a caring government, and many of our supporters do not understand why we are resisting a claim which they see as obviously just’. Since the DHSS had already accepted, privately, that the compensation principle was sound, ‘political considerations favour an early announcement … rather than waiting for many months, during which the pressure will build up and the vaccination programme … further damaged’.^72^ To give the government confidence, it was also becoming increasingly clear that respected medical evidence supported the pertussis vaccine.^73^

The Cabinet resolved to accept the general principle of compensation for victims of vaccine damage in order to restore faith in the vaccination programme. Partially, this was to ‘mollify’ Ashley and the Association.^74^ But it was also designed to play to the wider public. The belief was that by accepting the compensation principle, it would allay the fears of parents by showing that if something went wrong the state would protect them. It was also seen as a sign of strength and confidence. The government was explicitly stating that it was sure that there were so few cases that it was willing to compensate parents even if they could not definitively prove that vaccines were the sole cause of their child’s disability.^75^ On the other hand, it was possible that such action would bring attention to those rare cases, and give parents cause for concern.^76^ On balance, the government chose to acquiesce to the principle of the Association’s demands, and produce a solution that was financially affordable and would not open the government up to competing claims for no-fault compensation from other interest groups.^77^

The government engineered a public exchange of correspondence between Lord Pearson and James Callaghan, orchestrated by Ennals and Lord Chancellor Frederick Elwyn-Jones.^78^ On 6 June 1977, Callaghan wrote:

My ministerial colleagues and I are greatly concerned by the small, but tragic, number of cases in which vaccination against serious childhood diseases may have caused damage to the children concerned. … It would therefore go far to relieve the anxieties and concern of myself and colleagues, and to restore public confidence, if you were able to assure me that the Commission will be dealing specifically with the problem of vaccine damage and to give an indication of your thinking at this stage.^79^

To which Pearson responded:

I can readily give you the assurance you seek. … We see it as a particular part of a very difficult field with which our Report will have to deal, but we have all reached the conclusion that some kind of financial assistance should be made available for very serious injury resulting from vaccination recommended by a public health authority.^80^

The Association saw Pearson as a victory. While Pugh’s report had been seen as too tame, Pearson reaffirmed many of the core arguments, especially the one made in the *BMJ* in 1973—that is, that if the government was going to encourage all children to be vaccinated on public health grounds, it should also compensate those rare cases of damage that followed.^81^ Although the report only briefly covered vaccination policy (six pages out of 545), it acknowledged that almost every expert voice on the matter agreed that there was a moral case for compensation. ‘Nobody argued in the contrary sense.’^82^

The Government was forced to produce legislation quickly as the 1979 General Election was looming.^83^ Callaghan had already ordered that the scheme should be planned in the background to ensure it could be brought to the House as quickly after Pearson’s publication as possible.^84^ This meant that the drafting was essentially complete, and cross-party support for the Bill ensured that the passage through the Houses of Parliament became a formality.^85^ The resulting Act showed some very clear choices on the part of the DHSS which cannot be explained outside of the realm of disability policy. As the full title states, this was:

An Act to provide for payments to be made out of public funds in cases where severe disablement occurs as a result of vaccination against certain diseases or of contact with a person who has been vaccinated against any of those diseases.^86^

‘Severe disablement’ is, obviously, a reference to disability; the definition was based on long-standing medico-legal practice that had only recently been reaffirmed with the 1975 expansion of disability benefits. Thus, a person qualified for payment if they ‘[suffered] disablement to the extent of 80 per cent. or more, assessed as for the purposes of section 57 of the Social Security Act 1975’.^87^ The idea of ‘percentage of disablement’ came from the Industrial Injuries and War Pensions schemes from before the Second World War. The Disability Alliance favoured this system as a way of determining payments based on need for all disabled people, though in this respect they differed from DIG’s wider National Disability Income Scheme.^88^ The concept is, however, rooted in older medical definitions of disability. Percentage of disablement was designed to determine the effects on an adult being able to find employment ‘of a kind which apart from that injury, disease or deformity would be suited to his age, experience and qualifications’.^89^ The degree of disablement was measured against ‘a person of the same age and sex whose physical and mental condition is normal’ by a medical practitioner, who would provide a written assessment for the social security authorities.^90^ Transposing this concept onto children was not unheard of, despite the potential difficulties in applying such measures of disablement.^91^ By choosing 80 per cent—a generally accepted level of ‘severe disablement’ that would also be used when Severe Disablement Allowance was introduced in 1984—the government also made a decision that only the ‘most in need’ would receive benefit.^92^ This was a traditional tactic in restricting access to new benefits, with the DHSS and the Treasury often wary of opening the door to an avalanche of claims, and a seemingly exponential rise in public expenditure over time.^93^

It remains clear that the Vaccine Damage Payments Act 1979 could not have operated without the legal framework of disability that had been established over decades of legislation. Indeed, the restrictions on access were defended by reference to the supposed improvements in disability policy over the past decade. The Act was originally intended as an interim measure—the Labour government specifically brought it to Parliament with the caveat that once final recommendations on vaccines safety from JCVI were available (and Person had been fully digested) that there would be follow-up legislation to create a more comprehensive Act.^94^ This was in part a defence of the relatively low sum of money available: £10,000 was not considered enough by campaigners to truly cover the costs of caring for a severely disabled child over its lifetime. Yet this was also countered by reference to recent improvements in the general state of disability benefits.^95^ After the general election, Margaret Thatcher’s Conservative government used the defence that it planned to improve life for all disabled people and was not willing to give more special treatment to a group that already benefited hugely over other equally disabled people.^96^ Once again, the Act and its implementation were rooted in disability policy, legally and politically.

## The Significance of the Act

By looking at the historical and political context of the Vaccine Damage Payments Act, we can see more clearly why this specific piece of legislation was passed. Winning the medical argument through the Medical Research Council and JCVI evidence was not enough. A political statement needed to be made that accorded with public opinion and concern over the vaccination programme in general. The provisions contained within the Act were relatively cheap, a welcome relief in the economic circumstances. Initial estimates predicted only around 300 to 500 initial claims, followed by 14 to 70 claims per year thereafter. On the basis of £25,000 lump-sum payments, this would have cost around £10 million, and then £350,000 to £1,750,000 per annum.^97^ In the end, only £10,000 was awarded to each of 349 children in 1979 and 255 in 1980, before the claim rate fell significantly.^98^ It was also seen, to quote Ennals, as ‘one essentially of political judgement’. Vaccination was not compulsory in Britain, and it would be possible to argue that these cases affected the tiniest of minorities.^99^ Still, if the Association’s campaign had continued, Ennals believed that ‘the vaccination programme can be got going again’. It was ‘vital’ that it were, ‘because of the possibility of an outbreak of poliomyelitis this summer—an event for which many people would lay responsibility at the Government’s door’.^100^ The Act, and pronouncements leading to it, were part of a specific political response to a particular public threat.

The Association’s success was due to a number of factors, many of which are seen as typical of the campaigning landscape at the time. The use of more professionalised research, exploitation of media coverage and the creation of key allies in positions of power greatly aided the parents’ cause.^101^ The relationship between Rosemary Fox and Jack Ashley MP was central to this. Ashley had experience in precisely this sort of campaign for medical compensation following thalidomide. Cabinet spoke about the Association and Ashley in the same breath, referring to ‘Mr Ashley’s campaign’.^102^ Recent developments in disability policy cannot be ignored in explaining how these issues came to be recognised by the public as worthy of discussion. Social security payments were seen at this time as logical responses to social injustices; and disabled people were seen as worthy recipients of new benefit schemes. There were also a number of parliamentarians able to articulate these points. In many ways, the Association was unlike DIG and the Disability Alliance for its dogged focus on one specific benefit for a special medical case. However, it clearly benefited from many of the successes of those organisations, drawing on their tactics and building on their political arguments.

For it should be noted that The Association won the moral argument, and won it early. The only scientific debate to be won was to prove that vaccine damage existed. It managed to provide hundreds of potential examples, enough to spread doubt among the public and to win support for a compensation scheme. Pugh’s report, coupled with newspaper coverage of the medical doubts of Dick, Stewart and Wilson made vaccine damage a fact. It took the government three years to publicly announce that it accepted the thrust of the Association’s argument, as a direct result of the public pressure Fox and her allies had generated.^103^ This cannot be separated from the wider medical scare, but it reemphasises Drakeford and Butler’s claims that scandals have to be articulated and pursued.^104^ No person or body ever provided enough evidence to overturn the initial MRC trials; and two years later the completed review by the JCVI reaffirmed the medical establishment’s position.^105^ Without the wider focus on the political pressures of the period, we cannot explain why this particular medical debate became a full-blown ‘scandal’.

By establishing that there was a policy problem and generating the political will to rectify it, a solution needed to be found.^106^ Developments in disability benefits over the decade provided the bureaucratic tools for this. Since the late 1960s, the DHSS had been planning for a number of disability benefits. Even after the Social Security Benefits Act 1975, it had continued to be involved in developing a scheme for disabled drivers to purchase cars.^107^ Alfred Morris had established an Interdepartmental Group on Disablement within government, and the Sharp Report, Silver Jubilee Committee and Committee on Restrictions Against Disabled People were considering social rights issues with regard to access to businesses and services.^108^ In short, the British government had created the tools necessary for dealing with disability issues, both in the form of a bureaucratic apparatus for investigating policy solutions and the legal precedents of previous schemes. As we have also seen, concerns surrounding thalidomide had led to the introduction of medical issues in the Pearson Report. Thus, even if the decline in public support for vaccination is seen as a medical issue, the response cannot be explained outside the disability and social security politics of the late 1970s.

And yet, it must be stressed that most of the other disability organisations and campaigners of the period wrote very little about vaccine damage compensation. Disability studies activists, where they have written historical analyses of the 1970s, have focused on the battles they themselves fought. Since many of them were involved in DPOs such as the Union of the Physically Impaired Against Segregation and the British Council of Organisations of Disabled People, such legislation was neither part of their remit, nor was it a core constituent of the wider struggle for disabled people’s civil rights.^109^ Moreover, while the scheme is still running, very few disabled people received payments as a proportion of all the disabled people in the United Kingdom.^110^ Even at the time, organisations such as the Disability Alliance had covered Pearson in great detail and submitted evidence; but it had focused on industrial injuries compensation, not mentioning vaccination at all.^111^ Vaccine damage, then, occupies an interesting analytical hinterland, being both rooted in the disability politics and policy of the 1970s, while seemingly ignored as a disability issue by many of its contemporaries. This again stresses the need to look beyond solely medical readings of the pertussis vaccine scare. It also should make historians aware of the need to acknowledge that medical definitions of disability—while rejected by social model advocates as politically illegitimate—offer a useful lens for understanding and framing the decisions and attitudes of institutions in the past.

## Conclusions

The Association’s success quickly turned sour. Between 1974 and 1977, it won the moral argument for compensation, and legislation soon followed. After 1977, however, the focus shifted back towards public health. JCVI had warned of a potential whooping cough epidemic for 1978 or 1979, and when it hit, the Association and Jack Ashley took much of the blame.^112^ Dr Tony Smith in the *The Times* argued that while the press should have been more responsible in providing a balanced review of the evidence, it was the attention drawn by the Association that had caused the controversy.^113^ The JCVI and Office of Population Censuses and Surveys went further, arguing that the Association was to blame for scaring parents.^114^ Ashley was forced to refute at the time and many years later that he opposed vaccination, making it very clear that he and his colleagues supported the national programme and believed declining vaccination rates were worrisome.^115^ Such was the volatility of public opinion on the matter that in the summer of 1978 the government reported a shortage of whooping cough vaccine owing to the rush from parents who had previously opted out.^116^ In some ways, this marked a new medical scandal—not surrounding the failure of government protection against vaccine damage, but of its failure to protect against infectious disease. As Baker has shown, the targets of opprobrium were not a complacent medical establishment, but those who undermined the vaccination programme through scaremongering.^117^

The Vaccine Damage Payments Act was just a part of the government’s response to the pertussis vaccine scare. Ostensibly, it was designed to restore faith in the vaccination programme, allowing the state to resume its protective policies against infectious disease. But the specific form of this legislation and the way in which the campaigns for compensation were run owed a lot to the context of disability and social security developments over the course of the 1970s. The government had to act in such a way that took note of the economic conditions of the time, as well as public attitudes towards medical risk, vaccination and notions of the role of the state in protecting and providing for disabled people. Yet there is a paradox here for historians. Despite being seen as largely a matter of medical and public health policy, the responses to the pertussis scare were rooted in the context of disability and social security policy of the 1970s. It is clear that medical historians need to pay closer attention to the disability issues; and by the same token, disability historians can learn much from re-examining the Act using the skills they have developed over the past 20 years. This will begin to provide a wider view of the pertussis vaccine scare as a social phenomenon, and not just one of crisis within the public health profession.

## Footnotes

1 Dorothy Porter and Roy Porter, ‘The Politics of Prevention: Anti-vaccinationism and Public Health in Nineteenth-century England’, *Medical History*, 1988, 32, 231–52. Other investigations in smallpox and the Vaccination Acts include E. P. Hennock, ‘Vaccination Policy Against Smallpox, 1835–1914: A Comparison of England with Prussia and Imperial Germany’, *Social History of Medicine*, 1998, 11, 49–71; Ann Clark, ‘Compliance with Infant Smallpox Vaccination Legislation in Nineteenth-century Rural England: Hollingbourne, 1876–88’, *Social History of Medicine*, 2004, 17, 175–98; Michael Bennett, ‘Jenner’s Ladies: Women and Vaccination against Smallpox in Early Nineteenth-Century Britain’, *History*, 2008, 93, 497–513.

2 Anne Borsay, *Disability and Social Policy* (Basingstoke: Palgrave Macmillan, 2005). Again, recent studies have investigated the lives of children, disability campaigners and how war veterans were ‘rehabilitated’. See: Julie Anderson, *War, Disability and Rehabilitation in Britain* (Manchester: Manchester University Press, 2011); Michael Mantin, ‘Educational Experiences of Deaf Children in Wales: The Cambrian Institution for the Deaf and Dumb, 1847–1914’ (unpublished PhD thesis, Swansea University, 2012); Jameel Hampton, ‘Discovering Disability: The General Classes of Disabled People and the Classic Welfare State, 1948–1964’, *Historian*, 2013, 75.

3 Porter and Porter, ‘The Politics of Prevention’; Nadja Durbach, *Bodily Matters: The Anti-Vaccination Movement in England, 1853–1907* (Durham, NC: Duke University Press, 2004); Nelson Marie Clark and John Rogers, ‘The right to die? Anti-vaccination activity and the 1874 smallpox epidemic in Stockholm’, *Social History of Medicine*, 1992, 5, 369–88.

4 See: Anne Hardy, ‘Straight Back to Barbarism: Anti-typhoid Inoculation and the Great War, 1914’, *Bulletin of the History of Medicine*, 2000, 74, 265–90; Jane Lewis, ‘The Prevention of Diphtheria in Canada and Britain 1914–1945’, *Journal of Social History*, 1986, 20, 163–76.

5 Notable exceptions concern Bacillus Calmette-Guérin (BCG), Heaptitis B policy, and a growing interest in polio. Linda Bryder, ‘“We shall not find salvation in inoculation”’, *Social Science & Medicine*, 1999, 49, 1157–67; Jennifer Stanton, ‘What Shapes Vaccine Policy?’, *Social History of Medicine*, 1994, 7, 427–46; Gareth Williams, *Paralysed with Fear: The Story of Polio* (Basingstoke: Palgrave Macmillan, 2013); Ulrike Lindner and Stuart S. Blume, ‘Vaccine Innovation and Adoption: Polio Vaccines in the UK, the Netherlands and West Germany, 1955–1965’, *Medical History*, 2006, 50, 425–46.

6 G. Amirthalingam, S. Gupta and H. Campbell, ‘Pertussis Immunisation and Control in England and Wales, 1957 to 2012: A Historical Review’, *Euro Surveillance*, 2013, 18; Maria A. Riolo, Aaron A. King and Pejman Rohani, ‘Can Vaccine Legacy Explain the British Pertussis Resurgence?’, *Vaccine*, 2013, 31, 5903–8.

7 Chris T. Bauch and Samit Bhattacharyya, ‘Evolutionary Game Theory and Social Learning Can Determine How Vaccine Scares Unfold’, *PLoS Computational Biology*, 2012, 8, 1–12; Rachel Casiday, ‘Risk Communication in the British Pertussis and MMR Vaccine Controversies’, in Peter Bennett, Kenneth Calman, Sarah Curtis and Denis Fischbacher-Smith, eds, *Risk Communication and Public Health* (Oxford: Oxford University Press, 2010), 129–46; Rachel Casiday, ‘Risk and Trust in Vaccine Decision Making’, *Durham Anthropology Journal*, 2005, 13.

8 Jeffrey P. Baker, ‘The Pertussis Vaccine Controversy in Great Britain, 1974–1986’, *Vaccine*, 2003, 21, 4003–10; Department of Health and Social Security, Committee on Safety of Medicines, and Joint Committee on Vaccination and Immunisation, *Whooping Cough* (London: HMSO, 1981).

9 Virginia Berridge, *Public Health in History* (Maidenhead: Open University Press, October 2011), 213–16; Virginia Berridge, ‘History Matters?’, *Medical History*, 2008, 52, 311–26; Ben Goldacre, *Bad Science* (London: Harper Perennial, 2009). For histories of social welfare provision, see: Bernard Harris, *The Origins of the British Welfare State: Society, State and Social Welfare in England and Wales, 1800–1945* (Basingstoke: Palgrave Macmillan, 2004); Howard Glennerster, *British Social Policy, 1945 to the Present*, 3rd edn (Oxford: Blackwell, 2007); David Gladstone, ‘Renegotiating the Boundaries: Risk and Responsibility in Personal Welfare since 1945’, in Helen Fawcett and Rodney Lowe, eds, *Welfare Policy in Britain: The Road from 1945* (Basingstoke: Macmillan, 1999), 34–51.

10 Mark Drakeford and Ian Butler, *Scandal, Social Policy and Social Welfare* (Bristol: Policy Press, 2005).

11 Mark Drakeford and Ian Butler, ‘Everyday Tragedies: Justice, Scandal and Young People in Contemporary Britain’, *The Howard Journal of Criminal Justice*, 2007, 46, 219–35.

12 John W. Kingdon, *Agendas, Alternatives and Public Policies*, 2nd edn (New York: Longman, 1995); Gareth Millward, ‘Invalud Definitions, Invalid Responses: Disability and the Welfare State, 1965–1995’ (unpublished PhD thesis, London School of Hygiene & Tropical Medicine, 2014).

13 The DHSS had created the post of Minister for the Disabled in 1974, while governments since the mid-1960s had dedicated much administrative time to the question of disability benefits and access to services. See Gareth Millward, ‘Social Security Policy and the Early Disability Movement—Expertise, Disability, and the Government, 1965–77’, *Twentieth Century British History*, 2015, 26, 274–97.

14 For a historiographical review, see: Julie Anderson and Ana Carden-Coyne, ‘Enabling the Past: New Perspectives in the History of Disability’, *European Review of History*, 2007, 14, 447–57, and other articles in this special edition; Anne Borsay, ‘History and Disability Studies: Evolving Perspectives’, in Nick Watson, Alan Roulstone and Carol Thomas, *Routledge Handbook of Disability Studies* (Abingdon: Routledge, 2012), 324–35; Catherine J. Kudlick, ‘Disability History: Why We Need Another “Other”’, *The American Historical Review*, 2003, 108, 763–93.

15 Rodney Lowe, *The Welfare State in Britain since 1945* (Basingstoke: Palgrave Macmillan, 2005); Glennerster, *British Social Policy*; Derek Fraser, *The Evolution of the British Welfare State* (Basingstoke: Palgrave Macmillan, 2009).

16 See in particular: Anne Borsay and Pamela Dale (eds), *Disabled Children: Contested Caring, 1850–1979* (London: Pickering & Chatto, 2012); David M. Turner and Kevin Stagg (eds), *Social Histories of Disability and Deformity* (Abingdon: Routledge, 2006); Nick Watson (ed.), *Disability: Major Themes in Health and Social Welfare* (Abingdon: Routledge, 2008); Sonali Shah and Mark Priestley, *Disability and Social Change: Private Lives and Public Bodies* (Bristol: Policy Press, 2011).

17 Helen Bolderson, *Social Security, Disability and Rehabilitation* (London: Jessica Kingsley, 1991); Borsay, *Disability and Social Policy*; Julie Anderson, *War, Disability and Rehabilitation*; Hampton, ‘Discovering Disability’.

18 Ben Curtis and Stephen Thompson, ‘“A Plentiful Crop of Cripples Made by all this Progress”: Disability, Artificial Limbs and Working-class Mutualism in the South Wales Coalfield, 1890–1948’, *Social History of Medicine*, 2014, 27, 708–27; Alistair Ritch, ‘English Poor Law Institutional Care for Older People: Identifying the “Aged and Infirm” and the “Sick” in Birmingham Workhouse, 1852–1912’, *Social History of Medicine*, 2014, 27, 64–85; Heli Leppälä, ‘Duty to Entitlement: Work and Citizenship in the Finnish Post-war Disability Policy, early 1940s to 1970’, *Social History of Medicine*, 2014, 27, 144–64; Gwen A. Parsons, ‘The Construction of Shell Shock in New Zealand, 1919–1939: A Reassessment’, *Social History of Medicine*, 2013, 26, 56–73; Laura L. Phillips, ‘Gendered Dis/ability: Perspectives from the Treatment of Psychiatric Casualties in Russia’s early Twentieth-century Wars’, *Social History of Medicine*, 2007, 20, 333–50; Beth Linker, ‘Feet for Fighting: Locating Disability and Social Medicine in First World War America’, *Social History of Medicine*, 2007, 20, 91–109; and passim.

19 For thalidomide see: Jack Ashley, *Acts of Defiance* (London: Reinhardt, 1992); The Sunday Times, *Suffer the Children: The Story of Thalidomide* (New York: Viking Press, 1979); Mary Wilkinson, *Defying Disability* (London: Jessica Kingsley, 2009), 35–56; David Mason, *Thalidomide* (London: Allen and Unwin, 1976); Louise Medus, *Laughing and Loving—A Thalidomide Survivor’s Story* (Pembroke Dock: Accent Press, 2009). For vaccine damage see: Rosemary Fox, *Helen’s Story* (London: John Blake, 2006); Wilkinson, *Defying Disability*, 35–56; Ashley, *Acts of Defiance*.

20 Jameel Hampton, ‘Disabled People and the Classic Welfare State’ (Unpublished PhD thesis, University of Bristol, 2011).

21 Claire Sewell, ‘“If one member of the family is disabled the family as a whole is disabled”: Thalidomide Children and the Emergence of the Family Carer in Britain, c. 1957–1978’, *Family and Community History* (2015), 18, 37–52.

22 Baker, ‘The Pertussis Vaccine Controversy’.

23 See The National Archives, Kew (hereafter TNA) MH 154/1053, ‘“Society should compensate for brain damage”’, *Birmingham Post*, 26 June 1973 [page numbers omitted].

24 Mary McCormack, ‘The Hazards of Health’, *The Guardian*, 3 August 1973, 11. See also Fox, *Helen’s Story*.

25 Hampton, ‘Disabled People and the Classic Welfare State’; Campbell and Oliver, *Disability Politics*.

26 Since 1994, the Spastics Society has been called Scope. See: *ibid.*; Pat Thane, ‘Voluntary Action in Britain since Beveridge’, in Melanie Oppenheimer and Nicholas Deakin, eds, *Beveridge and Voluntary Action in Britain and the Wider British World* (Manchester: Manchester University Press, 2011), 121–34.

27 Disablement Income Group, *Creating a National Disability Income* (London: DIG, 1972); Hampton, ‘Disabled People and the Classic Welfare State’; Millward, ‘Social Security Policy and the Early Disability Movement’.

28 Disability Alliance, *Poverty and Disability* (London: Disability Alliance, 1975); Howard Glennerster, ‘Peter Townsend’, *Oxford Dictionary of National Biography* (Oxford: Oxford University Press, 2013).

29 Campbell and Oliver, *Disability Politics*, 55; Millward, ‘Social Security Policy and the Early Disability Movement’.

30 This was first properly articulated in Oliver, *Politics of Disablement*. However, its roots are deeper. See Paul Hunt, *Stigma* (London: G. Chapman, 1966); The Disability Archive, University of Leeds: Union of the Physically Imapaired Against Segregation and The Disability Alliance, *Fundamental Principles of Disability <*http://disability-studies.leeds.ac.uk/files/library/UPIAS-fundamental-principles.pdf*>*, accessed 13 August 2015; Victor Finkelstein, *Attitudes and Disabled People: Issues for Discussion* (New York: International Exchange of Information in Rehabilitation, 1980); Tom Shakespeare, *Disability Rights and Wrongs* (Abingdon: Routledge, 2006).

31 Victor Finkelstein’s story about the only non-wheelchair user in a world of wheelchair users is a good illustrative example. See: Victor Finkelstein, ‘Phase 2: Discovering the Person in “Disability” and “Rehabilitation”’, *Magic Carpet*, 1975, 27, 31–8.

32 Millward, ‘Social Security Policy and the Early Disability Movement’.

33
*Parliamentary Debates* (Commons), 767, 24 July 1969, 2158; *ibid*., 846, 14 November 1972, 96; *ibid.*, 881, 21 November 1974, 1558.

34 Eda Topliss and Bryan Gould, *A Charter for the Disabled* (Oxford: B. Blackwell & M. Robertson, 1981).

35 Alfred Morris and Arthur Butler, *No Feet to Drag: Report on the Disabled* (London: Macmillan, 1972); Derek Kinrade, *Alf Morris: People’s Parliamentarian: Scenes from the Life of Lord Morris of Manchester* (London: National Information Forum, 2007), esp. 155–82.

36 He was, for instance, the Labour vice president of DIG. See Ashley, *Acts of Defiance*; Beth Capper, *A Celebration of the Work of the APPDG* (London: RADAR, 2008), 12.

37 Fox, *Helen’s Story*, 57–61.

38 See correspondence with Ashley in TNA: BN 13/360; and also TNA: BN 124/20, Meeting on the Vaccine Damage Payments Scheme, 16 June 1981.

39 Millward, ‘Invalid Definitions, Invalid Responses’.

40 Fox, *Helen’s Story*, 88.

41
*Parliamentary Debates* (Commons) 847, 29 November 1972, 446.

42 See: Hampton, ‘Disabled People and the Classic Welfare State’; Derek Kinrade, *Alf Morris* (London: National Information Forum, 2007), 207–18; Ashley, *Acts of Defiance*; The Sunday Times, *Suffer the Children*; Medus, *Laughing and Loving*.

43 Colin Pearson, *Royal Commission on Civil Liability and Compensation for Personal Injury*, vol. 1 (Cmnd. 7054–I) (London: HMSO, 1978), specifically quotes thalidomide cases as one of the reasons for its appointment.

44 A. Robens, *Safety and Health at Work, Report of the Committee* (Cmnd. 5034) (London: HMSO, 1972).

45 Sewell, ‘Thalidomide Children and the Emergence of the Family Carer’.

46 Anon., ‘Help for Victims of Immunizations’, *Br Med J.*, 1973, 1, 758.

47 Virginia Berridge, Martin Gorsky and Alex Mold, *Public Health in History* (Maidenhead: Open University Press, 2011), 195–210.

48
*Parliamentary Debates* (Commons) 766, 20 June 1968, 1294–5; *ibid.*, 795, 11 February 1970, 1356–7; *ibid.*, 914, 8 July 1976, 1979; *ibid.*, 957, 7 November 1978, 110–2W; *Parliamentary Debates* (Lords) 317, 7 April 1971, 376–7.

49 See in particular Fox’s autobiography in which she details the campaign from her perspective. Fox, *Helen’s Story*.

50 Whiteley and Winyard, *Pressure for the Poor: The Poverty Lobby and Policy Making* (London: Methuen, 1987); Matthew Hilton, James McKay, Nicholas Crowson and Jean-François Mouhot, *The Politics of Expertise: How NGOs Shaped Modern Britain* (Oxford: Oxford University Press, 2013), esp. 133–5; Matthew Hilton, Nick Crowson, Jean-François Mouhot and James McKay, *A Historical Guide to NGOs in Britain: Charities, Civil Society and the Voluntary Sector since 1945* (Basingstoke: Palgrave Macmillan, 2012), 122–5.

51 ‘Vaccination against Whooping-cough’, *Br Med J.*, 1956, 2, 454.

52 This had been a core complaint in the interview with the *Guardian*. Mary McCormack, ‘The Hazards of Health’, *The Guardian*, 3 August 1973, 11.

53 J. V. T. Gosling and J. H. Moseley, ‘To Jab or Not to Jab?’, *The Guardian*, 20 August 1973, 9.

54 M. Kulenkampff, J. S. Schwartzman and J. Wilson, ‘Neurological Complications of Pertussis Inoculation’, *Archives of Disease in Childhood*, 1974, 49, 46–9.

55 McCormack, ‘The Hazards of Health’, 11.

56 This created ripples across the developed world. See: Per Axelsson, ‘The Cutter Incident and the Development of a Swedish Polio Vaccine, 1952–1957’, *Dynamis*, 2012, 32, 311–28; Alison Day, ‘“An American Tragedy”. The Cutter Incident and its Implications for the Salk Polio Vaccine in New Zealand 1955–1960’, *Health & History: Journal of the Australian & New Zealand Society for the History of Medicine*, 2009, 11, 42–61.

57 As of January 1977, Fox claimed to have 281 cases of damage, of which 182 were blamed on pertussis. Hugh Herbert, ‘Parents Gain Ground in Medical Fight’, *The Guardian*, 6 January 1977, 6. See also: Fox, *Helen’s Story;* DHSS *et al.*, *Whooping Cough*.

58 William Breckon, ‘A Vaccinating Question’, *The Guardian*, 29 August 1973, 9.

59 G. Stewart, ‘Vaccination against Whooping-cough’, *Lancet*, 1977, 1, 234–7; ‘Rush for Cough Vaccine “could do harm”, *The Guardian*, 19 December 1977, 2; Baker, ‘The Pertussis Vaccine Controversy’.

60 See, e.g., correspondence between Fox and the Heath government in Kew, London: The National Archives, TNA: MH 154/1053; and with the Wilson/Callaghan governments in TNA: PIN 35/549.

61 Fox, *Helen’s Story*, 67–70. Early Day Motion 70 (1974–75), for example, had been signed by ‘more than 50’ MPs by 3 December 1974: ‘That this House is concerned at the lack of statistics concerning vaccine-damaged children: believes that their case for compensation is at least as just as those children suffering as a result of the thalidomide tragedy; and demands an immediate investigation into the problem’, *Parliamentary Debates* (Commons) 882, 3 December 1974, 1514–26.

62 For questions from Astley see: *ibid.*, 867, 17 January 1974, 172–5W; *ibid.*, 914, 1 July 1976, 277–8W; *ibid.*, 924, 17 January 1977, 73–4W and passim. For questions from Adley see: *ibid*., 918, 2 November 1976, 547–8W; *ibid.*, 923, 20 December 1976, 240–59; *ibid.*, 925, 7 February 1977, 575–7W and passim.`

63 ‘The Departments’ referred to the Department of Health and Social Security (and its predecessors in England and Wales) and the Scottish and Northern Ireland Offices which had devolved responsibility for health. Parliamentary Commissioner for Administration, *Sixth Report, Whooping Cough Vaccination* (London: HMSO, 1977), HC571 (1976–77), 3.

64 Peter Hillmore, ‘MP Picks 4 Vaccine Victims for Fight’, *The Guardian*, 25 January 1976, 6.

65 Parliamentary Commissioner for Administration, *Sixth Report*, 17–18, 22.

66 ‘Vaccination Campaign Shelved’, *The Guardian*, 22 November 1977, 4; ‘Minister Defers Campaign to Encourage Vaccination’, *The Times*, 22 November 1974, 2; ‘Vaccine Campaign Launched Soon’, *The Guardian*, 8 February 1978, 5.

67
*Parliamentary Debates* (Commons) *925*, 8 February 1977, 1227–39.

68 ‘Bringing the Law up to Scratch’, *The Guardian*, 7 August 1974.

69 Fox, *Helen’s Story*, 88–90. See also TNA: MH 154/1057.

70 TNA: CAB 129/195/14, Merlyn Rees, Vaccine Damage, 29 April 1977, 1.

71 Hugh Herbert, ‘Which? Backs Vaccine Injury Compensation’, *The Guardian*, 13 January 1977, 7.

72 TNA: CAB 129/195/16, David Ennals, Payment for Vaccine Damaged Children, 2 May 1977, 1.

73 ‘Medicine: Doubts over Vaccine Damage’, *The Times*, 26 February 1977, 14; Hugh Herbert, ‘“No cause for alarm” as Whooping Cough Spreads’, *The Guardian*, 26 April 1978.

74 TNA: CAB 128/61/18, CM(77) 18th Conclusions, Cabinet minutes, 5 May 1977 10.30am, 10.

75 TNA: CAB 129/195/14, Vaccine Damage; CAB 129/195/16, Payment for Vaccine Damaged Children; *Parliamentary Debates* (Commons) *925*, 8 February 1977, 1227–39.

76 TNA: CAB 129/195/14, Vaccine Damage, 1. Similar views were expressed in a *Guardian* editorial: ‘Either Way, the Children still Die’, *The Guardian*, 12 February 1977.

77 TNA: CAB 128/61/18, Cabinet minutes, 5 May 1977, 8–11.

78 See TNA: BN 120/10, esp. Lord Elwyn-Jones to James Callaghan, 20 May 1977; Cabinet Office, Meeting between Lord Chancellor, Secretary of State for Social Services and Chief Secretary, Treasury, held 19 May 1977.

79 TNA: BN 120/10, James Callaghan to Lord Pearson, 6 June 1977.

80 TNA: BN 120/10, Lord Pearson to James Callaghan, 9 June 1977. This exchange was reinforced by a statement by Ennals in the House of Commons: *Parliamentary Debates* (Commons) 933, 14 June 1977, 240–1.

81 For allegations that the Pugh report was too tame see: Gillian Linscott, ‘“Frail” Vaccine Report Disappoints Parents’, *The Guardian*, 28 October 1972, 2.

82 Pearson, *Royal Commission*, para. 1397, 296.

83 An election had to be called by October as it had been five years since the previous one, but it would be brought forward by a vote of no confidence in March 1979. *Parliamentary Debates* (Commons) 965, 28 March 1979, 461–590.

84 TNA: CAB 128/61/18, Cabinet minutes, 5 May 1977, 10–11.

85 TNA: BN 13/360; The Bill got its second reading on 5 February and received Royal Assent on 22 March, days before the no-confidence vote. *Parliamentary Debates* (Commons) 962, 5 February 1979; *ibid.*, 964, 22 March 1979, 1760.

86 Vaccine Damage Payments Act 1979.

87
*Ibid.*, c. 1, para. 4. In Northern Ireland it references the Social Security (Northern Ireland) Act 1975.

88 Disability Alliance, Poverty and Disability; DIG, Creating a National Disability Income.

89 Definition of ‘disablement’, used in all subsequent Acts until the Disability Discrimination Act 1995. Disabled Persons (Employment) Act 1944, c. 1.

90 Social Security Act 1975, Schedule 8.

91 Paul Abberley, ‘Counting Us Out: A Discussion of the OPCS Disability Surveys’, *Disability, Handicap & Society*, 1992, 7, 139–155; Glenn T. Fujiura and Violet Rutkowski-Kmitta, ‘Counting Disability’, in Albrecht *et al.*, eds, *Handbook of Disability Studies*, 69–96.

92 For the Severe Disablement Allowance levels of disablement see: Health and Social Security Act 1984, c. 11.

93 Millward, ‘Invalid Definitions, Invalid Responses’. See also Stone’s work on how disability schemes tend to grow as pressure builds from within: Deborah A. Stone, *The Disabled State* (Philadelphia: Temple University Press, 1984); Deborah A. Stone, ‘Physicians as Gatekeepers’, *Public Policy*, 1979, 27, 227–54.

94 JCVI published its recommendations in 1981, along with data on the effectiveness of the vaccine. See DHSS *et al.*, *Whooping Cough*; Anon., ‘Efficacy of Pertussis Vaccination in England’, *Br Med J.*, 1982, 285, 357–39; R. Alderslade, Department of Health and Social Security, and Middlesex Hospital Medical School, *National Childhood Encephalopathy Study* (London: Middlesex Hospital Medical School, 1981).

95
*Parliamentary Debates* (Commons) 962, 5 February 1979, 32–86.

96 TNA: BN 124/20, Norman Fowler to Fox, 4 March 1983; Margaret Thatcher to Fox, (?)20 April 1982; TNA: PIN 35/549, Gerard Vaughan to Fox, 22 September 1980; *Parliamentary Debates* (Commons) 977, 22 January, 163W.

97 TNA: CAB 129/195/14, Vaccine Damage, Annex.

98 While the value of the award would be scaled up at points over the proceeding decade to combat the effects of inflation, the number of claims remained relatively low. A total of 74 successful claims were made in 1981; 42 in 1983; 29 in 1984; 26 in 1985; 15 in 1986 and 10 up to December 1987. See *Parliamentary Debates* (Commons) 124, 18 December 1987, 931W.

99 TNA: CAB 128/61/18, Cabinet minutes 5 May 1977, 8–11.

100 TNA: CAB 129/195/16, Payment for vaccine damaged children, 1.

101 See: Whiteley and Winyard, *Pressure for the Poor*.

102 CAB 128/61/18, Cabinet minutes 5 May 1977, 8.

103 See Ennals’ arguments in CAB 12/195.16, Payment for vaccine damaged children.

104 Drakeford and Butler, ‘Everyday Tragedies’.

105 DHSS *et al.*, *Whooping Cough*; Baker, ‘The Pertussis Vaccine Controversy’.

106 See Kingdon’s work on policy making and agenda setting: Kingdon, *Agendas, Alternatives and Public Policies.*

107 TNA: BN 59/75.

108 For examples of the Interdepartmental Group on Disablement and of cabinet sub-committee discussions Morris led, see TNA: CAB 134/3845; CAB 134/4036; CAB 134/4235; MH 154/848; and passim. Evelyn Adelaide Sharp chaired the Sharp Report, published as *Mobility of Physically Disabled People* (London: HMSO, 1974). The Silver Jubilee Committee was chaired by Peter Large and its report published as ‘Can Disabled People Go Where You Go?’: Report by the Silver Jubilee Committee on Improving Access for Disabled People (London: HMSO, 1979). See also: Committee on Restrictions Against Disabled People, *Report* (London: HMSO, 1982).

109 Campbell and Oliver, *Disability Politics*; Michael Oliver and Colin Barnes, *The New Politics of Disablement* (Basingstoke: Palgrave Macmillan, 2012).

110 Only 843 payments had been made in total from the Vaccine Damage Payments scheme up to December 1987. By contrast, over one million people were claiming invalidity benefit at any one time in 1987/88. See *Parliamentary Debates* (Commons) 124, 18 December 1987, 931W. There were an average of 1,067,000 claimants at any one time of invalidity benefit, the main National Insurance unemployment benefit for disabled people, in the financial year 1987/88. Department of Work and Pensions, *Benefit Expenditure Tables*, 2013 <http://statistics.dwp.gov.uk/asd/asd4/expenditure_tables_Budget_2013.xls>, accessed 12 June 2013.

111 The Disability Alliance’s files in the Peter Townsend Collection at the University of Essex cover this period in great detail. See especially: Peter Townsend Collection, University of Essex, Colchester: 79.03, ‘A note about the Pearson Commission Report’, Peter Townsend 16 June 1978; 77.02, Disability Alliance Steering Group minutes, 29 June 1978, 1.

112 Reports of declining vaccination rates and increased risk of epidemic were published across the winter of 1977/78. See for example: ‘Whooping Cough Vaccinations Fall’, *The Guardian*, 23 November 1977, 4; ‘Whooping Cough Could Sweep Britain’, *The Guardian*, 10 December 1977, 24; ‘The Omens so Far Are Bad—These Could be the First Ripples of a Whooping Cough Wave’, *The Guardian*, 25 January 1978, 9.

113 Dr Tony Smith, ‘Clearing the Doubts over Whooping Cough’, *The Times*, 18 August 1978, 12.

114 Melanie Phillips, ‘Row over Cause of Whooping Cough Outbreak’, *The Guardian*, 9 August 1978, 3.

115 Ashley, *Acts of Defiance*. See also *Parliamentary Debates* (Commons) 962, 5 February 1979, 33; *Parliamentary Debates* (Lords) 551, 12 January 1994, 125. Fox and the Association were also keen to emphasise that they were not anti-vaccination, but were looking for more informed choice and compensation for those injured:‘Mary McCormack, ‘The Hazards of Health’, 11; ‘Bringing the Law up to Scratch’, *The Guardian*, 7 August 1974.

116 ‘Whooping Cough Vaccine “almost run out”’, *The Times*, 16 December 1977, 2.

117 Baker, ‘The Pertussis Vaccine Controversy’, esp. 4006.

